# Critical Influence of the Processing Route on the Mechanical Properties of Zirconia Composites with Graphene Nanoplatelets

**DOI:** 10.3390/ma14010108

**Published:** 2020-12-29

**Authors:** Ángela Gallardo-López, Carmen Muñoz-Ferreiro, Cristina López-Pernía, Emilio Jiménez-Piqué, Felipe Gutiérrez-Mora, Ana Morales-Rodríguez, Rosalía Poyato

**Affiliations:** 1 Departamento de Física de la Materia Condensada, ICMS, CSIC-Universidad de Sevilla, Avda. Reina Mercedes s/n, 41012 Sevilla, Spain; cmunoz7@us.es (C.M.-F.); cristinalopez@us.es (C.L.-P.); fegumo@us.es (F.G.-M.); amr@us.es (A.M.-R.); 2 Barcelona Research Center in Multiscale Science and Engineering, Universidad Politécnica de Cataluña, Avda. Eduard Maristany 10-14, 08019 Barcelona, Spain; Emilio.Jimenez@upc.edu; 3 Departament of Materials Science and Engineering, Universidad Politécnica de Cataluña, Avda. Eduard Maristany 10-14, 08019 Barcelona, Spain; 4 Instituto de Ciencia de Materiales de Sevilla, ICMS, CSIC-Universidad de Sevilla, Avda. Américo Vespucio 49, 41092 Sevilla, Spain; rosalia.poyato@icmse.csic.es

**Keywords:** yttria tetragonal zirconia polycrystals (YTZP), graphene nanoplatelets (GNP), planetary ball milling (PBM), spark plasma sintering (SPS), instrumented hardness, elastic modulus, flexure strength

## Abstract

Graphene-based nanostructures, used as potential reinforcement in ceramic composites, have a great tendency to agglomerate. This requires the use of homogenization techniques during the powder processing, posing the need to evaluate how these techniques affect the microstructure and the mechanical properties of the resulting composites. The influence of the processing route on the properties of 3YTZP (3 mol % yttria tetragonal zirconia polycrystals) ceramic composites with 10 vol % cost-effective GNP (graphene nanoplatelets) has been addressed. Four different powder processing routines combining ultrasonic powder agitation (UA) and planetary ball milling (PBM) in wet and dry media have been used and all the composites were densified by spark plasma sintering (SPS). The mechanical properties at room temperature in the macroscale have been assessed by Vickers indentations, four-point bending tests and the impulse-echo technique, while instrumented indentation was used to measure the hardness and Young’s modulus at the nanoscale. The application of dry-PBM enhances greatly the mechanical and electrical isotropy of the composites, slightly increases the hardness and lowers the elastic modulus, independently of the application of UA. The combination of UA and dry-PBM enhances the flexure strength by 50%, which is desirable for structural applications.

## 1. Introduction

Ceramics are still attractive structural and functional materials due to their unique resistance to high temperatures, resistance to corrosion and inertness [1]. Tetragonal zirconia, in particular 3YTZP, stands out from advanced ceramics due to its exceptional fracture toughness [2], with applications in the aerospace industry [3] and in the field of dental ceramics [4,5,6] also due to its biocompatibility [7]. In the last decade, the addition of graphene-based nanostructures to ceramics has attracted the attention of the scientific community [8,9] pursuing reinforcement effects in the resulting composites or enhanced functional properties such as electrical conductivity. From the applications point of view, the improvement of the mechanical properties of ceramics is crucial for structural purposes [10,11], while the achievement of a high electrical conductivity opens the door to advanced machining techniques such as electro discharge machining (EDM) to allow the fabrication of miniaturized and complex ceramic shapes for parts or tools [12].

Although many studies have been devoted to investigating the mechanical properties of graphene nanoplatelets (GNP)–ceramic composites [13,14,15,16,17], only a few of them focus on 3YTZP ceramic matrix composites [18,19]. The comparison of mechanical behavior between composites with similar GNP content is not straightforward since key aspects such as the processing and sintering techniques and conditions used and the quality and size of the graphene filler [20] can lead to very different microstructures. The grain size of the ceramic, the GNP distribution into the matrix and the nature of the GNP–ceramic interface are microstructural issues which strongly influence the mechanical properties. Besides, the use of different experimental mechanical testing approaches can also give different results. That is why studies like the one proposed here, which compare several of these aspects independently (processing routine, experimental testing method) while maintaining others unaltered (filler characteristics and content, sintering technique), are very necessary. In addition, although there are studies focused on instrumented hardness, giving results of hardness and elastic moduli of these GNP–ceramic composites on the nanoscale [15,21,22,23] and others which have addressed the macroscopic Vickers [24] or Knoop [25] hardness and elastic moduli values [16,18,26], the present study provides complementary results of the mechanical properties on the micro and the macroscale. It is also important to provide mechanical results on different specimen orientations to take into account the possible anisotropy of these composites [16,27,28,29]. This is especially necessary when they have been sintered by a technique involving uniaxial pressure during the sintering process, which increases the anisotropy caused by the high aspect ratio of these 2D-like fillers. The importance of comparing different processing routines or sintering conditions in GNP/ceramic composites to see the impact on the mechanical properties has been addressed by different authors [30,31,32]. However, only a few compare different experimental mechanical testing techniques [18]. Using different mechanical testing techniques on the same composites is important to allow a reasonable comparison between the varied mechanical results existing in the literature.

The aim of this work is to assess the mechanical performance of ceramic composites with 10 vol % cost-effective GNP (graphene nanoplatelets) densified by SPS from powders subjected to different processing routines, involving either ultrasonic wet powder mixing, planetary ball milling in wet/dry media or combinations of both mixing techniques. These powder processing techniques alter the GNP size, crystallinity and distribution into the ceramic matrix, as it was reported in a previous work [33]. In order to evaluate the effect of these parameters on the room-temperature mechanical properties of the 3YTZP-GNP composites, their hardness and elastic modulus were measured at the nanoscale by instrumented hardness tests in order to take into account the elastic relaxation upon unloading, which is particularly important in ceramics, and at the macroscale by Vickers indentation and the impulse-echo technique, to assess the mechanical homogeneity and isotropy of the composites. Finally, four-point bending tests were also used to estimate the flexure strength and the flexure modulus.

## 2. Materials and Methods

3YTZP-10 vol % GNP composites were fabricated from commercial powders: TZ-3YB-E ceramic powder with 40 nm particle size supplied by Tosoh Europe B.V, Amsterdam, Netherlands and N006-P graphene nanoplatelets with <5 μm lateral size and 10–20 nm thickness supplied by Angstrong Materials, Dayton, OH USA. Four different powder processing routines were followed: (i) UA: wet powder mixing by ultrasonic agitation, (ii) UA-W-PBM: wet powder mixing by ultrasonic agitation followed by wet planetary ball milling, (iii) UA-D-PBM: wet powder mixing by ultrasonic agitation followed by dry planetary ball milling and (iv) D-PBM: dry powder mixing by planetary ball milling. The procedure was covered in detail in a previous paper [33].

Elemental microanalysis was used to assess the GNP (equivalent to the C) content in the composite powders after processing, by means of a TruSpec CHNS micro LECO elemental micro-analyzer (Centro de Investigación, Tecnología e Innovación de la Universidad de Sevilla, CITIUS, Sevilla, Spain).

The composite powder mixtures were densified using SPS with the conditions detailed in [33]: 1250 °C sintering temperature, 5 min hold time and 75 MPa in vacuum.

The sintered composites with cylindrical shape (15 mm diameter × 3 mm thickness) were lapped manually to remove the graphite adhered during sintering, and then grinded to achieve parallel faces. Density was estimated by the Archimedes’ method [34]. Theoretical density was calculated by the rule of mixtures [35] from the values 2.2 g/cm^3^ (GNP) and 6.05 g/cm^3^ (3YTZP) given by the suppliers.

Standard Vickers indentations (Vickers Duramin indenter, Struers, Copenhagen, Denmark) on perpendicular mirror-polished surfaces (parallel and perpendicular to the composites’ sintering axis) were performed to estimate the composites’ hardness at the macroscale and to check for anisotropy effects. The hardness values were calculated averaging ten indentations on each surface, using the equation H_V_ (GPa) = 1854.4 P/D^2^ [36] where P is the applied load (1.96 N for 10 s), and D the average diagonal of the imprint (in μm). Hardness at the nanoscale was estimated by means of nanoindentation tests using a Nanoindenter XP (MTS System Corporation, Eden Prairie, MN, USA) with a fully calibrated Berkovich tip and a continuous stiffness measurement module, as described in [37]. The hardness (H) and Young’s modulus (E) were calculated as a function of penetration depth using the method proposed by Oliver and Pharr [38].

The impulse excitation technique was used to estimate the Young’s modulus (E) at the macroscale by measuring the acoustical response of the cylindrical-shaped composites to a short mechanical pulse with Sonelastic^®^ equipment and software (ATCP Physical Engineering, Ribeirão Preto, Brazil).

Four-point bending tests were carried out for the composites processed using dry planetary ball milling (UA-D-PBM and D-PBM). Four bars (15 × 2.5 × 2 mm^3^) were tested for each composite. The bar surface in tension was polished up to 1 µm diamond paste, while the edges were chamfered with 10 and 2 µm diamond paste. To adapt to the specimen’s small size, a special lab-designed miniature 4-point bending assembly [39] was attached to a universal 1165 INSTRON (Norwood, MA, USA) machine. The bending tests were performed at room temperature with 0.5 mm/min span speed. These tests allowed a rough estimate of the flexural resistance and the flexural modulus to be made.

## 3. Results and Discussion

**Figure 1:**
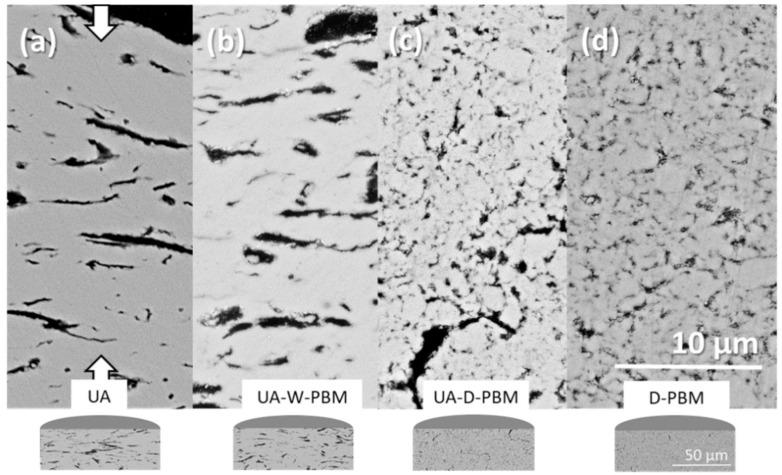
Backscattered electrons (BSE)-SEM images of the polished cross sections of the composites prepared from graphene nanoplatelets (GNP) and 3YTZP powders mixed by different routines: (**a**) ultrasonic agitation, (**b**) ultrasonic agitation followed by wet planetary ball milling, (**c**) ultrasonic agitation followed by dry planetary ball milling and (**d**) dry planetary ball milling. The same scale has been used for the micrographs (**a**–**d**). Compression axis during spark plasma sintering (SPS) is indicated in (**a**) by arrows. A schematic representation of the sample section (not to scale) is given for each composite.

The microstructural characterization of the composites by microanalysis, X-ray diffraction (XRD), Raman spectroscopy, laser granulometry and scanning electron microscopy (SEM) is covered in detail in a previous paper [33]. The most relevant difference in the composites prepared using these four different processing approaches was the size and degree of dispersion of the GNP in the ceramic matrix, which can be visualized in Figure 1.

The results of the GNP morphology characterization performed from the analysis of SEM micrographs from polished cross sections of the composites in a previous study [33] are summarized in Table 1. D_GNP_ corresponds to the GNP aggregates lateral size, D_max_ is the maximum lateral size value observed, d_GNP_ is the thickness, A.R._GNP_ is the aspect ratio (D_GNP_/d_GNP_) and, finally, d_3YTZP_ is the ceramic grain size, corresponding to the planar diameter. The parameters that have been statistically obtained are followed by their corresponding standard deviations (s.d.). These results are shown because they are important microstructural parameters to explain the mechanical behavior of the different composites.

**Figure 2:**
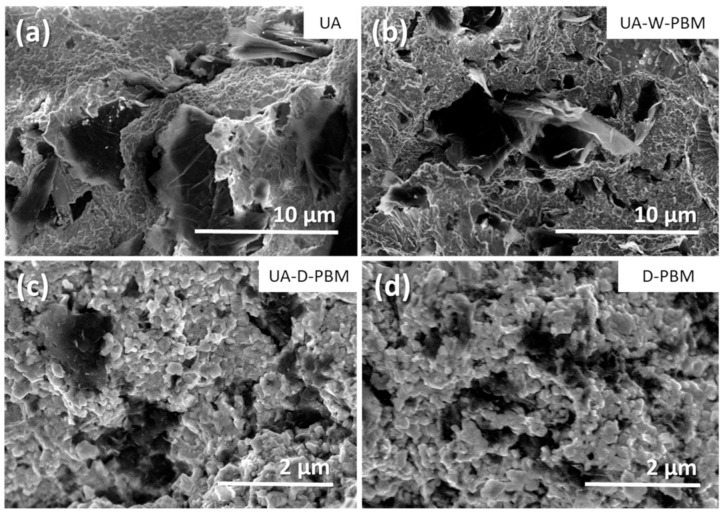
Secondary electrons (SE)-SEM images of the fracture surfaces of the composites prepared from GNP and 3YTZP powders mixed by different routines: (**a**) ultrasonic agitation, (**b**) ultrasonic agitation followed by wet planetary ball milling, (**c**) ultrasonic agitation followed by dry planetary ball milling and (**d**) dry planetary ball milling.

We have to point out that the GNP size is slightly reduced when planetary ball milling is applied to the powder suspension (wet media, W-PBM) and considerably reduced when planetary ball milling is applied to the powders (dry conditions, D-PBM). The dry PBM promotes both the fragmentation and the exfoliation of the GNP, and also reduces the aspect ratio of the GNP, producing more isotropic microstructures. Although the GNP morphological parameters for both composites using dry PBM are similar, the most remarkable difference is the maximum GNP size, lower in the D-PBM one, which points to a more effective size reduction with this homogenization routine. This effect was more clearly observed from the dynamic laser scattering performed to the powders. The GNP distribution and morphology can also be visualized in the backscattered electrons (BSE)-SEM micrographs of Figure 1 and at a higher magnification with more detail in the secondary electrons (SE)-SEM micrographs of Figure 2. In this Figure 2, the size, shape and distribution of the GNP within the ceramic matrix can be distinguished. The four composites have been ordered according to the GNP maximum size (D_max_) for easier comparison. Differences in the GNP surface can also be observed, with crumpling and wavy surfaces in the composites from powders subjected to UA and mostly flat surfaces in the D-PBM composite. The ceramic grain size is lower and more homogeneous (lowest standard deviation) in the D-PBM composite, due to the smaller GNP size which allowed their enhanced distribution around the ceramic grains. It was concluded that the homogenization of the powder mixture by high-energy planetary ball milling in dry conditions without previous ultrasonication resulted in the composite with the best performance in terms of microstructural homogeneity and electrical conductivity [33].

### 3.1. Mechanical Characterization

#### 3.1.1. Hardness

The results of the hardness tests carried out at the nanoscale by instrumented indentation and at a larger scale by Vickers indentations on the composites prepared using the mentioned four different powder mixing routines are listed and compared in Table 2.

All the composites are above 95% of the theoretical density, and the small fluctuations on the experimental density values do not seem to influence the hardness results. Although all the composites had a 10 vol % nominal GNP content, the slight differences in the real GNP content detected by the microanalysis assessment of the composite powders can affect the mechanical properties of the dense materials.

**Figure 3:**
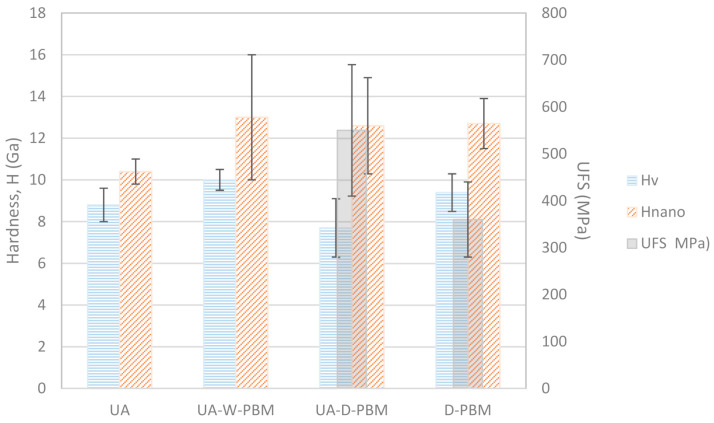
Vickers and nano-indentation hardness values (averaged for in i.p. -in plane- and c.s. –cross section- orientations) and ultimate flexure strength of the composites processed by the different techniques.

The composites prepared from powders mixed with dry planetary ball milling (D-PBM and UA-D-PBM) have similar hardness values and are more isotropic, especially the one which included ultrasonic agitation (UA-D-PBM). The composite D-PBM is the most homogeneous, with the smallest standard deviation. The rest of the composites are more anisotropic, being harder when the indenter hits the cross-section surface, where the GNP lie edgewise. This behavior has been already reported for 3YTZP-GNP composites from powders processed by ultrasonic agitation [40,41], and can be attributed to low energy mechanisms such as delamination and sliding of the graphene layers, which activate when the indentation load is applied on the perpendicular plane to the sintering axis. These mechanisms were also reported in bulk sintered multilayered graphene flakes [42]. Another clear influence of the processing routine is that the composite processed by D-PBM is harder than the one processed only by ultrasonic agitation, even when its GNP content is higher. A higher GNP content would lead to lower hardness for composites processed with the same technique. Comparing the experimental techniques in the different scales, the results show that in all the composites, the use of instrumented hardness at the nanoscale results in higher values than the Vickers hardness obtained using macroscopic measurement methods, as it has also been observed in similar composites with lower GNP content [41]. The hardness values averaged for the two orientations have been represented in Figure 3, for easier comparison between the different mechanical testing methods. The flexure strength has also been plotted.

#### 3.1.2. Elastic Moduli

The nano-indentation method was the only one which was carried out on two different orientations of the specimens to obtain the Young’s modulus. According to this method, the Young’s modulus in the i.p.—in plane, where the GNP in the anisotropic composites lie on their main plane—is very similar for all the composites (see Table 3), no matter which processing technique was followed. The results in the c.s. –cross section- were systematically higher than in the i.p., (except for the isotropic UA-D-PBM composite), following the same trend that was found in the hardness results. This dependence on the orientation of the Young’s modulus has been also reported for silicon nitride ceramic composites with graphene fillers [43], although an opposite trend was observed. However, a similar dependence to our trend (higher values in c.s.), was reported for the shear modulus, G. The small differences encountered in the cross-section values for the different composites could be related to their slight differences in the GNP content (stiffest composite for lowest GNP content, and the lowest modulus in the composite with the highest GNP content). Since the length scale of these measurements is very low, the reason for this correlation could be that the probability of the indenter (of the same magnitude order than the lateral size of the GNP) hitting in a GNP with a low elastic modulus in the c.s. increases when the GNP content rises. The difference is less remarkable when the GNP lie on their main plane, cause in that case the probability of the indenter tip hitting the ceramic rises when the GNP lateral size decreases, resulting in higher hardness and elastic modulus values for the composites with smaller GNP, which correspond to the ones which include D-PBM in their processing routine [33]. Since the GNP from the composite D-PBM are the smallest in size (see Table 1), and of the same order than the nano-indentation length scale, the probability of the indenter tip hitting GNP is smaller in this composite, resulting in higher hardness and elastic modulus values when using this nano-scale technique.

The elastic modulus estimation by the echo-pulse technique is based on the response to a mechanical pulse which impinged on the i.p. surface of the composites, so the results should be compared to the i.p. elastic modulus measured by nano-indentation. According to the impulse-echo technique, the composites with the lowest elastic modulus were UA-D-PBM and the highest elastic modulus was exhibited by the composites UA. Taking into account that the GNP which were only subjected to sonication during the mixing step are larger and less damaged, this result is in line with the results from Seiner et al. [44], who found that a higher exfoliation degree the graphene filler results in a decrease in the elastic modulus in ceramic composites with a Si_3_N_4_ matrix. In the present study, it is also observed that the more aggressive the mixing procedure, with wavier and smaller GNP, the lower the elastic modulus. Our results also indicate that the lateral size reduction of the filler is also related to the decrease of the elastic modulus. The Young’s modulus obtained for D-PBM and UA-D-PBM by this method are very similar.

The four-point bending tests were carried out on rectangular bars whose surface in tension–compression corresponded to the i.p. face, where the GNP in the anisotropic composites lie on their main plane, so the results are related to this configuration. The four-point bending tests were only performed to the composites which displayed a more homogeneous microstructure, the ones that had undergone dry planetary ball milling (D-PBM and UA-D-PBM). The composites D-PBM showed a lower flexure modulus than UA-D-PBM, but taking into account the large experimental uncertainties, the values can be considered similar.

Comparing the different mechanical testing methods, the highest elastic modulus values are those measured by nano-indentation, following the same trend than the hardness results. These values are higher due to the different way of measuring. Hardness in nano-indentation is Meyer’s hardness, which use the projected contact area for measuring hardness. In Vickers, the contact area is used instead, so values are always lower. In addition, Young’s modulus can be slightly overestimated due to possible effects of the Poisson ratio in the relationship between stiffness and Young’s modulus [45,46].

**Figure 4:**
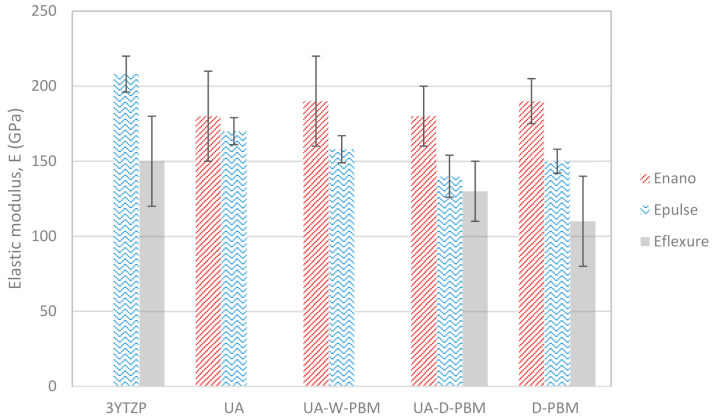
Comparison of the average values of elastic modulus measured by nano-indentation, echo-pulse technique and bending tests for the 3YTZP ceramic matrix and the composites with 10 vol % GNP processed by the different techniques.

Besides, the results of hardness and elastic modulus for these composites at the nanoscale are fairly dependent on the GNP filler size, and thus they are not so representative of the overall behavior of the material. It is sensitive in a different scale and could be useful to detect the interfacial properties between the filler and the matrix. The flexure modulus values obtained by means of the stress-strain curves recorded from four-point bending tests are the lowest elastic moduli of the three mechanical testing techniques. Although ideally the flexure modulus and the Young’s modulus of a solid should have the same value, they may differ in real conditions due to the non-ideality of the material or of the test conditions [47]. In our case, the composites might experiment plastic deformation due to sliding between GNP plates or GNP interfaces with zirconia, and the bar-like specimens might contain flaws caused during the machining. Besides, the equivalence of the flexural and Young’s moduli assumes equivalent compressive and tensile moduli, an assumption that is not usually true in the case of ceramics. Our results, which have been plotted for comparison in Figure 4, show that this mechanical testing method produces the largest uncertainties. Therefore, in view of the elastic moduli values obtained, and given that the impulse-echo method is (i) non-destructive, (ii) based on the macroscopical response of the specimen, and (iii) does not involve many machining steps, we find it very suitable and its results may be considered the most reliable for these ceramic–GNP composites.

#### 3.1.3. Flexure Strength

The flexure strength was only evaluated in the most homogeneous composites, the ones from powders processed using dry planetary ball milling. The results of the stress to failure (UFS) measured in the specimens from different batches are listed in Table 4.

The results of UFS indicate that the composites which included ultrasonic dispersion of the GNP prior to the dry planetary ball milling exhibit a 50% increase in flexure strength compared to the ones which had not been subjected to ultrasonic agitation. This was a surprising finding since we expected very similar flexure strength results. The reason for this discrepancy can be that the application of ultrasonic agitation effectively disperses the GNP, breaking agglomerates and thus enhances their lubricating effect during the milling with the 3YTZP ceramic powder. This can explain the larger GNP size in the UA-D-PBM composites compared to the D-PBM ones. The larger GNP size is associated with an increase in the flexure strength. The larger GNP anchors strongly to the ceramic composite, effectively bridging cracks [48], while reinforcing mechanisms provided by the GNP, such as crack deflection, crack branching, crack bridging, etc., are considerably reduced when their lateral size is very low [43,48].

## 4. Conclusions

In this work, the hardness and elastic modulus have been estimated in 3YTZP–GNP composites to assess the influence of four different mixing approaches during the powder processing. The mechanical characterization has been carried out with different testing methods at the nano- and the macroscale to compare the results and establish their validity range for these composites.

The non-destructive impulse-echo technique gives reliable results for the Young’s modulus in these composites. Nano-indentation also produces reliable and consistent results. Differences in the obtained values are attributed to the different ways of measuring the mechanical properties.

The use of dry planetary ball milling (D-PBM and UA-D-PBM) during the powder processing compared to milder mixing procedures produced slightly harder composites with an isotropic response to indentation. However, the more aggressive the mixing procedure, the smaller the GNP size and the lower the elastic modulus.

The composites which had included in the powder processing ultrasonic agitation previous to dry planetary ball milling (UA-D-PBM) presented a 50% higher flexure strength than those which were processed by direct dry milling (D-PBM). The use of UA previous to D-PBM enhances the GNP dispersion into the ceramic powder, increasing the GNP lubricating effect during the milling and therefore limiting its size reduction. The larger GNP can provide better crack inhibition mechanisms.

## Figures and Tables

**Table 1:** Morphological parameters of the GNP and grain size of the 3YTZP ceramic matrix measured for each composite [33]. The first column indicates the different powder processing techniques described in the experimental part: (i) UA: wet powder mixing by ultrasonic agitation, (ii) UA-W-PBM: wet powder mixing by ultrasonic agitation followed by wet planetary ball milling, (iii) UA-D-PBM: wet powder mixing by ultrasonic agitation followed by dry planetary ball milling and (iv) D-PBM: dry powder mixing by planetary ball milling.

Composite	D_GNP_ (µm)	s.d. (µm)	Dmax (µm)	d_GNP_ (µm)	s.d. (µm)	A.R._GNP_	s.d.	d_3YTZP_ (µm)	s.d.
**UA**	2.3	2.0	16.5	0.6	0.4	3.7	2.5	0.25	0.11
**UA-W-PBM**	1.5	1.5	12.1	0.35	0.21	4.3	2.7	0.30	0.12
**UA-D-PBM**	0.32	0.30	2.6	0.16	0.09	2.0	1.0	0.22	0.10
**D-PBM**	0.39	0.22	1.6	0.18	0.08	2.2	0.8	0.18	0.07

**Table 2:** Vickers hardness and instrumented hardness on each orientation of the composite (in-plane and cross section) and average values. The real GNP content and the relative density of the composites are also indicated.

Composite	GNP (vol %)	ρ_r_ (%)	H (GPa) Vickers		H (GPa) Nano		H (GPa) (Average)	
			In-Plane	c.s.	In-Plane	c.s.	Vickers	Nano
**UA**	9.5	99.7 ± 0.2	8.0 ± 0.9	9.5 ± 1.8	10 ± 3	11.0 ± 1.6	8.8 ± 0.8	10.4 ± 0.6
**UA-W-PBM**	8.6	98.4 ± 1.1	9.5 ± 1.3	10.4 ± 0.7	12 ± 3	15 ± 3	10 ± 0.5	13 ± 3
**UA-D-PBM**	12.8	98.3 ± 0.5	7.4 ± 1.4	7.9 ± 0.6	12.6 ± 2.1	12.6 ± 2.3	7.7 ± 1.4	12.6 ± 2.3
**D-PBM**	10.1	95.9 ± 1.3	9.4 ± 0.9	9.4 ± 0.9	11.5 ± 1.2	13.9 ± 0.5	9.4 ± 0.9	12.7 ± 1.2

**Table 3:** Young’s modulus from nano-indentation experiments (E_nano_), macroscopical results of the Young’s modulus from impulse-echo technique (E_pulse_) and flexure modulus from bending (E_flexure_) tests. Some values for the reference monolithic 3 mol % yttria tetragonal zirconia (3YTZP) are also included.

Specimen	E_nano_ (GPa) (In-Plane)	E_nano_ (GPa) (Cross-Section)	E_nano_ (GPa) Average	E_pulse_ (GPa) Average	E_flexure_ (GPa) Average
**3YTZP**	_	_	_	208 ± 12 [44]	150 ± 30
**UA**	170 ± 30	190 ± 13	180 ± 30	170 ± 9	_
**UA-W-PBM**	170 ± 16	220 ± 30	190 ± 30	158 ± 9	_
**UA-D-PBM**	174 ± 15	180 ± 20	180 ± 20	140 ± 14	130 ± 20
**D-PBM**	174 ± 10	204 ± 5	190 ± 15	150 ± 8	110 ± 30

**Table 4:** Density and ultimate flexure strength (UFS) of the composites from powder processed by ultrasonic agitation followed by dry planetary ball milling (UA-D-PBM) and dry planetary ball milling (D-PBM).

	UA-D-PBM		D-PBM	
	Relative Density (%)	Relative Density (%)	UFS (MPa)	UFS (MPa)
	95.7 ± 1.5	94.0 ± 0.5	273	361
	97.4 ± 0.4	98.1 ± 0.3	352	650
	97.3 ± 0.3	99.7 ± 0.3	379	606
	97.2 ± 0.3	99.1 ± 0.5	437	566
**Average**	**96.9 ± 0.9**	**98 ± 3**	**360 ± 80**	**550 ± 140**

## Data Availability

The data presented in this study is contained within the article.
